# S100B Protein, A Damage-Associated Molecular Pattern Protein in the Brain and Heart, and Beyond

**DOI:** 10.1155/2010/656481

**Published:** 2010-08-18

**Authors:** Guglielmo Sorci, Roberta Bianchi, Francesca Riuzzi, Claudia Tubaro, Cataldo Arcuri, Ileana Giambanco, Rosario Donato

**Affiliations:** Department of Experimental Medicine and Biochemical Sciences, University of Perugia, Via del Giochetto, 06122 Perugia, Italy

## Abstract

S100B belongs to a multigenic family of Ca^2+^-binding proteins of the EF-hand type and is expressed in high abundance in the brain. S100B interacts with target proteins within cells thereby altering their functions once secreted/released with the multiligand receptor RAGE. As an intracellular regulator, S100B affects protein phosphorylation, energy metabolism, the dynamics of cytoskeleton constituents (and hence, of cell shape and migration), Ca^2+^ homeostasis, and cell proliferation and differentiation. As an extracellular signal, at low, physiological concentrations, S100B protects neurons against apoptosis, stimulates neurite outgrowth and astrocyte proliferation, and negatively regulates astrocytic and microglial responses to neurotoxic agents, while at high doses S100B causes neuronal death and exhibits properties of a damage-associated molecular pattern protein. S100B also exerts effects outside the brain; as an intracellular regulator, S100B inhibits the postinfarction hypertrophic response in cardiomyocytes, while as an extracellular signal, (high) S100B causes cardiomyocyte death, activates endothelial cells, and stimulates vascular smooth muscle cell proliferation.

## 1. Introduction

S100 is a multigenic family of small (~10 kDa) Ca^2+^-binding proteins of the EF-hand type comprising 25 members exclusively expressed in vertebrates [1, 2]. In humans, the genes encoding S100A1-S100A16, S100A7L2, S100A7P1, and S100A7P2 map to chromosome 1q21, and the genes encoding S100A11P, S100B, S100G, S100P, and S100Z map to chromosomes 7q22-q31, 21q22, Xp22, 4p16, and 5q13, respectively [3]. With the exception of S100G which is a Ca^2+^-modulator protein involved in the buffering of cytosolic Ca^2+^, the members of this protein family are Ca^2+^ sensor proteins which once activated by Ca^2+^ interact with intracellular target proteins thereby regulating their activities. However, S100A10 is a constitutively activated protein involved in regulatory functions irrespective of the cytosolic Ca^2+^ level. It should be pointed out that: (1) the Ca^2+^-binding affinity of S100 proteins (as measured in vitro) is considerably lower than that of the universal, intracellular Ca^2+^ sensor protein, calmodulin [4]; (2) however, S100's Ca^2+^-binding affinity increases in the presence of target proteins (5) and/or, for some S100 members, Zn^2+^ [5, 6]; and (3) Ca^2+^-independent interactions have been described for certain S100 proteins [7]. Importantly, differently from the ubiquitous calmodulin, individual S100 proteins exhibit a cell-specific expression, and some S100 proteins exert both intracellular and extracellular regulatory activities [1, 8]. Moreover, with the exception of S100G which is monomeric, all other S100 proteins exist within cells as dimers (mostly homodimers, and in certain cases heterodimers), and some of the secreted/released S100 proteins form oligomers [1, 9, 10].

S100B was the first member of the S100 protein family to be identified. This protein is highly abundant in the brain where it localizes to astrocytes (which represent the most abundant source of S100B in absolute), although certain neuronal populations also appear to express it [1, 10]. S100B is also expressed in cells outside the brain, such as melanocytes, adipocytes, chondrocytes, Schwann cells, glial cells of the gastrointestinal apparatus, supporting cells of the adrenal medulla, dendritic cells, mature skeletal myofibers, skeletal muscle satellite cells, and arterial smooth muscle cells [1, 10–12]. Cardiomyocytes do not express S100B, but S100B becomes expressed in the cardiomyocytes surviving an infarction under the action of catecholamines [13–15].

S100B is constitutively secreted by astrocytes and its secretion can be regulated by a number of factors [10]. It is also secreted by adipocytes along with free fatty acids under the action of catecholamines [16]. Moreover, S100B is passively released from damaged and/or necrotic cells. The presence of S100B in the cerebrospinal fluid, serum, and amniotic fluid above threshold levels is used for diagnostic/prognostic purposes [17].

S100B exerts regulatory activities within cells and, once secreted/released, it acts as an extracellular signal. However, accumulating evidence suggests that intracellular regulatory activities of S100B differ substantially from its extracellular effects; that is, no unitary theory of intracellular and extracellular S100B's effects can be envisaged at present.

## 2. Intracellular S100B

As an intracellular regulator, S100B has been implicated in the regulation of protein phosphorylation, energy metabolism, the dynamics of cytoskeleton constituents (and hence, of cell shape and migration), Ca^2+^ homeostasis, and cell proliferation and differentiation [10]. The large variety of cell activities regulated by S100B can be explained by the high abundance of the protein in S100B-expressing cells and its cytoplasmic localization. Thus, the enhanced expression of S100B in melanoma cells has been suggested to be causally related to tumor progression given that S100B not only interacts with the tumor suppressor, p53, blocking its phosphorylation [18] but also downregulates p53 expression (with p53 in turn downregulating S100B expression) [19], and pharmacological blockade of S100B activity with pentamidine, a drug that disrupts S100B-p53 interactions [20], results in a significant tumor growth inhibition [21]. However, interaction with and/or downregulation of p53 might not be the sole mechanism whereby intracellular S100B stimulates cell proliferation. Indeed, intracellular S100B has been shown to stimulate proliferation and modulate cell differentiation via activation of PI3K and its downstream signaling pathways in neuronal and astrocytic cell lines [22, 23] and to modulate cell differentiation via activation of IKK*β*/NF-*κ*B in myoblast cell lines [12], and its induction by the so-called SOX trio in chondroblasts has been causally related to inhibition of chondrocyte differentiation [14]. Moreover, S100B positively regulates migration in astrocytes via activation of a Src/PI3K/RhoA/ROCK module [23]. Thus, intracellular S100B might serve the function of negatively regulating cell differentiation and stimulating proliferation and migration in cell lines. Whether S100B serves these functions during development and tissue regeneration remains to be established. A tight regulation of S100B expression appears to take place during neurogenesis, with relatively high S100B expression levels in neural progenitor cells as long as they are proliferating and migrating, followed by repression of S100B expression in coincidence with glial precursor cell differentiation and resumption of S100B expression in differentiated astrocytes [23, 24]. Interestingly, S100B appears to be required for MIO-M1 cells, a human Müller glia cell line, to form neurospheres (i.e., spherical aggregates of highly proliferating, round cells characterized by low adhesiveness to the substrate and the expression of transcription factors characteristic of neural stem cells [25]) [23]. Indeed, S100B knockdown in these cells has been shown to result in reduced neurosphere formation and proliferation and acquisition of an astroglial phenotype (unpublished results). Also, rat primary astrocytes transiently downregulate S100B expression when exposed to the differentiating agent, db-cAMP, and reexpress S100B at later stages of db-cAMP-induced differentiation [23]. In this case, as well as in the case of glioma cell lines induced to acquire a differentiated phenotype by serum starvation, reexpressed S100B firstly appears to be located at the origin of cell extensions in proximity of F-actin bundles [23]. These results are compatible with the possibility that: (1) S100B is required for neural progenitor cells to maintain stemness and migratory capacity; (2) transient repression of S100B expression is functionally associated with early steps of astrocytic differentiation; (3) persistence of S100B expression in neural progenitor cells might result in disturbances in neural cell differentiation [26, 27]; and (4) reexpression of S100B in differentiated astrocytes might be functionally linked to the maintenance of astrocytic processes as well as other cell activities (see above). The molecular mechanism regulating S100B expression in astrocytes depending on the developmental stage remains to be identified; preliminary evidence suggests that EGF signaling might cause downregulation of S100B expression in differentiating astrocytes [24]. Characteristically, S100B expression is enhanced in astrogliosis, a process consisting of proliferation and activation of astrocytes followed by their hypertrophy as observed after a brain insult that compromises brain tissue integrity or during chronic brain inflammatory states [28]. This raises the possibility that S100B might contribute to astrocyte reactivity following brain damage by favoring both the migration of activated astrocytes to the site(s) of damage and the formation and/or stabilization of F-actin cytoskeleton in astrocytic processes, likely via a Src/PI3K/RhoA/ROCK pathway and a Src/PI3K/Akt/GSK3*β*/Rac1 pathway [23], and possibly by regulating as yet unidentified intracellular activities via the Src/PI3K module. PI3K is known to play a regulatory role in inflammatory cells [29, 30], and astrocytes are active players in innate immunity in the brain [31]. Indeed, there is evidence that PI3K signaling might play an important role in both astrogliogenesis [32] and astrocytic activation in neuroinflammation [33–36]. Experimental evidence suggests that administration of arundic acid (ONO-2506), an agent suggested to inhibit S100B synthesis [37], in a rodent ischemia model results in inhibition of overexpression of S100B in astrocytes and the subsequent activation of signaling pathways in the peri-infarct area, in a reduction of delayed infarct expansion and in amelioration of neurologic deficits [38]. Conversely, after permanent middle cerebral artery occlusion in S100B transgenic (TG) mice, infarct volumes are significantly increased during the first postinfarct days and astrogliosis is enhanced compared with controls [39]. Moreover, S100B TG mice show increased susceptibility to perinatal hypoxia-ischemia [40], and overexpression of S100B has been shown to accelerate Alzheimer disease-like pathology with enhanced astrogliosis and microgliosis [41]. In this regard, association between elevated brain levels of S100B and several brain pathologies including Alzheimer disease is a well-established notion [10, 42]. Although in the aforementioned cases [38–41] it is difficult to distinguish between intracellular and extracellular effects of S100B, it is tempting to speculate that elevated levels of intracellular S100B might contribute to astrocyte activation during the course of brain damage and to astrogliosis. Yet, these putative effects of intracellular S100B appear to be counterbalanced by S100B extracellular effects in part (see below).

As mentioned earlier, cardiomyocytes do not express S100B in normal physiological conditions; however, S100B becomes expressed in cardiomyocytes surviving an infarction under the action of catecholamines and acts to inhibit the cardiomyocyte hypertrophic response with a mechanism that remains to be elucidated [13–15].

Genetic evidence has been presented that S100B exerts inhibitory effects on caffeine-induced rises in the free Ca^2+^ concentration in astrocytes, suggesting that S100B might act to reduce cytosolic Ca^2+^ concentration [43]. However, the molecular mechanism underlying this S100B effect has not been identified. On the other hand, acute infusion of S100B knockout (KO) mice with norepinephrine (NE) after a 28-day treatment with NE results in a significantly smaller increase in mean arterial pressure (MAP) compared to wild-type (WT) and S100B TG mice, with a tendency of S100B TG mice to respond with higher MAP values compared with WT mice [11]. Arterial smooth muscle cells (ASMCs) from S100B KO mice are less responsive to NE treatment compared with WT ASMCs due to either reduced Ca^2+^ mobilization from internal Ca^2+^ stores or reduced extracellular Ca^2+^ influx [11], pointing to a requirement of S100B for appropriate Ca^2+^ responses to NE. However, this does not apply to cardiomyocytes from S100B KO mice pointing to a dissociation between effects of S100B in cardiomyocytes and those in ASMCs in the same experimental setting (i.e., stimulation with NE) [11]. The molecular mechanism underlying S100B's ability to enhance cytosolic Ca^2+^ concentration in ASMCs also remains to be identified. The giant phosphoprotein AHNAK, which modulates L-type Ca^2+^ channels in response to *β*-adrenergic stimulation [44], interacts with phospholipase C*γ* and PKC-*α* increasing intracellular Ca^2+^ mobilization [45] and is expressed in smooth muscle cells and cardiomyocytes [46], is an S100B target protein [47]. Thus, AHNAK is a potential intermediate linking S100B to elevation of cytosolic Ca^2+^ levels [11]. However, additional intermediates appear to come into play because S100B-AHNAK interactions also occur in cardiomyocytes which do not require S100B for increasing cytosolic Ca^2+^ levels in response to NE, as mentioned earlier. Together, these results point to a differential ability of S100B to intervene in the regulation of the cytosolic Ca^2+^ concentration in activated cells depending on the cell type, suggesting that cell-specific intermediates might link S100B to regulators of cytosolic Ca^2+^ levels.

## 3. Extracellular S100B

### 3.1. S100B Acts as an Extracellular Signal in Brain, Vasculature and Heart

The first evidence for the presence of S100B outside neural cells was provided by Michetti et al. [48] who detected measurable amounts of S100B in the cerebrospinal fluid of patients with multiple sclerosis. S100B was ever since taken as a marker of brain disease [17]. Subsequently, Shashoua et al. detected S100B in the brain extracellular fluid [49], and later on, Van Eldik and Zimmer [50] demonstrated that an astrocyte cell line secreted S100B under conventional culture conditions. Secretion of S100B from astrocytes, which occurs via a noncanonical secretion route, was subsequently shown to be regulated by a number of factors/conditions, of which some enhance secretion (e.g., serotonin, lysophosphatidic acid, low levels of glutamate, forskolin, low extracellular Ca^2+^ and/or K^+^ levels, TNF-*α*, IL-1*β*, metabolic stress, serum deprivation, kainic acid, the neurotoxin 1-methyl-4-phenyl 1,2,3,6 tetrahydropyridine, natural antioxidants, and antipsychotic drugs) while some other reduce secretion (e.g., high levels of glutamate, glucose and K^+^, inhibition of Src kinase activity, cell confluence, Ca^2+^ channel blockers and gap junction inhibitors) [10, 51–53]. However, a rather small fraction of intracellular S100B is being secreted constitutively by astrocytes, and S100B secretagogues cause a 2-4-fold increase in secretion at most [10]. Given that the total brain S100B concentration amounts to 10–20 *μ*M; that the brain intercellular space is relatively narrow; and that a fraction of secreted S100B diffuses into the cerebrospinal fluid, the brain extracellular S100B concentration should amount to a few nM under normal physiological conditions. Yet, the brain S100B concentration outside cells might be several orders higher in case of astrocyte damage and/or necrosis due to a combination of passive release of the intracellular protein, defective clearance of the extracellular protein in consequence of inflammation, and/or Ca^2+^-induced formation of S100B oligomers [54, 55] and adhesion of S100B oligomers to the extracellular matrix.

**Figure 1:**
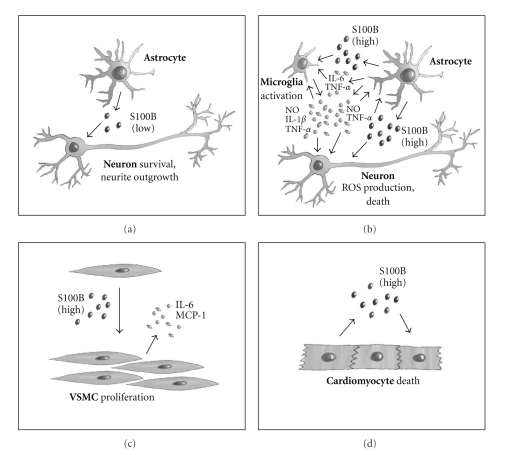
Schematic representation of extracellular effects of S100B in brain, heart, and vasculature. (a) At low concentrations, S100B promotes neuronal survival and stimulates neurite outgrowth via stimulation of RAGE signaling. (b) At high concentrations, S100B causes neuronal death both directly via excessive stimulation of RAGE signaling in neurons and indirectly via RAGE-dependent activation of microglia and astrocytes. (c) At high concentrations, S100B stimulates VSMC proliferation via RAGE activation. See text for details. (d) S100B released from necrotic cardiomyocytes kills nearby, surviving cardiomyocytes via RAGE activation.

The existence of an extracellular fraction of S100B was soon put in relation to effects of the protein on brain cells inasmuch as a neurite extension factor from bovine brain was identified as a disulfide-linked dimer of S100B [56]. Whereas early reports on extracellular S100B supported the possibility that the protein might function as a trophic factor towards neurons and astrocytes [10], the observation that elevated levels of S100B were present in the temporal lobe of patients with Alzheimer disease in conjunction with the presence of highly reactive S100B-positive astrocytes surrounding the neuritic plaques [57] raised the possibility that S100B might contribute to Alzheimer disease neuropathology. Thus, research on extracellular S100B proceeded ever since along two main directions, on the basis of the protein's dual role as a neurotrophic factor and as a neurotoxic factor. These apparently contradictory effects of S100B in the brain were shown to be dependent on the protein's concentration, with doses of S100B up to a few hundred nM being neurotrophic and higher doses being neurotoxic [42]. Indeed, S100B at high doses causes neuronal apoptosis both via a direct action on neurons [58] and via stimulation of nitric oxide (NO) release by astrocytes [59] (Figure 1(a)). Moreover, at high doses, and in the presence of cofactors (either bacterial endotoxin or interferon-*γ* [IFN-*γ*]), S100B enhances NO release by microglia [60, 61], the brain resident macrophages (Figure 1(b)). The finding that S100B can activate microglia, albeit at high doses, suggests that the protein might have a role in neuroinflammation, a possibility substantiated by other studies of effects of S100B on microglia and astrocytes [62–66] (Figure 1(b)).

A strong impulse to research on extracellular effects of S100B in the brain came from the observation that RAGE (receptor for advanced glycation end products), a multiligand receptor of the immunoglobulin superfamily expressed in several cell types including neurons during development as well as in activated inflammatory cells, transduces S100B's effects on endothelial cells and microglia [67]. Indeed, RAGE ligation by S100B on neurons has been shown to be responsible for both the protein's neurotrophic effects (at low S100B doses) via activation of a Ras/MEK/ERK/NF-*κ*B/Bcl-2 pathway and a Ras/Cdc42-Rac1 pathway, and pro-apoptotic effects (at high S100B doses) via hyperactivation of the Ras/MEK/ERK pathway and consequent overproduction of reactive oxygen species (ROS) [68] (Figure 1(b)). These latter results anticipated that: (1) extracellular S100B might affect any RAGE expressing cell; (2) the outcome of S100B action might be dependent on RAGE functionality and/or concentration; (3) the different outcome of S100B effects depending on the protein's concentration might be dependent on the number of RAGE molecules engaged on the cell surface and/or the physical state of S100B; and (4) the relatively high doses of S100B required for RAGE-dependent activation of inflammatory cells, compared with absence of effects at low doses, might reflect a differential ability of RAGE to recruit different intermediates linking the receptor to intracellular signaling pathways depending on the cell type, RAGE's physical state and/or the intensity/duration of RAGE stimulation.

As to point (1), S100B has been indeed used ever since as a generic RAGE agonist [8, 10]. However, RAGE-independent effects of low and high doses of S100B have been documented in the case of cultured myoblasts (which express RAGE) in differentiation medium [69–71], which raises the possibility that extracellular factors might regulate S100B/RAGE interactions. Indeed, recent evidence suggests that in high-density myoblast cultures S100B (up to 1 nM) induces the formation of a multimeric RAGE/S100B/bFGF/FGFR1 transcomplex in which bFGF/FGFR1 antimyogenic signaling is enhanced while RAGE promyogenic signaling is inhibited, as opposed to RAGE-dependent regulatory effects of S100B in low-density myoblast cultures (a condition in which S100B does not bind to bFGF/FGFR1 and thus fully activates RAGE) (F. Riuzzi, G. Sorci and R. Donato, submitted for publication).

As to point (2), in general no effects of S100B could be detected in neurons, astrocytes, and inflammatory cells that had been transfected with a dominant negative RAGE mutant (i.e., RAGE lacking the cytoplasmic and transducing domain) or in which RAGE expression had been knocked down (11). However, S100B has been shown to stimulate NO release from IFN-*γ*-treated microglia to the same extent in microglia that had been stably transfected with either full-length RAGE or dominant negative RAGE, but to a significantly larger extent in these cases compared with mock-transfected microglia [72]. This suggests that at least in the case of S100B-induced NO release by microglia, the RAGE extracellular domain might serve to concentrate S100B on the microglial cell surface thereby allowing S100B to potentiate IFN-*γ* effect. Whether S100B interacts with IFN-*γ* thereby potentiating IFN-*γ* effects remains to be established. Yet, at high doses S100B causes overproduction of ROS via activation of the NADPH oxidase complex in monocytes/macrophages in the absence of co-factors, with ROS in turn, activating Src tyrosine kinase which recruits a Ras/MEK/ERK1/2/NF-*κ*B pathway and a Rac1/Cdc42/MKK6/p38 MAPK/NF-*κ*B pathway with ensuing upregulation of IL-1*β*, TNF-*α*, COX-2 and iNOS expression [73, 74]. In these latter works, RAGE-signaling has been proposed, but not directly demonstrated to have a role in S100B effects. By contrast, at high doses S100B causes myoblast death via ROS overproduction, via a RAGE-independent mechanism that remains to be elucidated [70]. On the other hand, S100B/RAGE signaling-dependent RAGE induction and activation in neuronal cells might contribute to the protective effect of low S100B doses towards *β*-amyloid neurotoxicity and to amplification of *β*-amyloid neurotoxicity by high S100B doses [75].

As to point (3), preliminary evidence suggests that at high, but not low doses S100B chemoattracts microglia in a RAGE-dependent manner; however at low doses the protein does chemoattract RAGE-overexpressing microglia (R. Bianchi, Eirini Kastrisianaki, I. Giambanco, and R. Donato, submitted for publication). This suggests the possibility that at the levels found in normal physiological conditions brain extracellular S100B cannot affect microglia migration due to the very low, if any expression levels of RAGE in “resting” microglia, but the protein might contribute to chemoattract microglia at the beginning of a brain insult as a result of activation of microglia, a condition which is accompanied by induction of RAGE in these cells [76]. Thus, increasing the density of RAGE molecules on the microglial cell surface might cause S100B to switch from a neurotrophic factor to a proinflammatory factor. In this regard, it is known that RAGE is induced in a variety of cell types by RAGE-activating ligands [77, 78]. Moreover, recent evidence suggests that extracellular S100B exists in the form of octamers and higher-order multimers in the nonreducing, high Ca^2+^ conditions found in the extracellular fluid; that RAGE exists in the form of oligomers on the cell surface; and that ligand-induced oligomerization is required for RAGE to signal adequately [54, 55, 79]. Thus, the neurotoxic and proinflammatory effects of S100B might require the occurrence of high levels of S100B oligomers/multimers interacting with RAGE oligomers (and/or causing/enhancing RAGE oligomerization). Importantly, doses of S100B causing neuronal apoptosis do not cause microglial apoptosis, a difference which is likely to depend on the different scavenging ability of neurons and microglia towards oxidants. Microglia activation and chemotaxis are being usually considered in the context of neuroinflammation. However, it should be pointed out that microglia chemoattraction is not necessarily a dangerous and/or inflammation-related event; “resting” microglia are not exactly resting, microglia continuously patrolling the territory, exerting a protective action by virtue of their ability to keep the neuronal and astrocytic extracellular milieu clean, and likely resolving mild degree brain insults [80, 81]. According to these views microglia are active players in brain tissue homeostasis under normal physiological conditions, and thus (low) S100B's ability to chemoattract microglia might be beneficial in case of mild degree brain insults. However, whereas there is information about the increased susceptibility to perinatal hypoxia-ischemia and accelerated Alzheimer disease-like pathology with enhanced astrogliosis and microgliosis in a background of overexpressed S100B [82, 83], no information is available in a background of deletion of the S100B gene. Thus, whether or not is there any role of S100B in microglia-mediated brain tissue homeostasis in normal physiological conditions remains to be established.

As to point (4), RAGE engagement has been shown to result in the activation of several downstream signaling pathways [77, 78]. However, there appears to be no univocal set of signaling pathways that are being activated by RAGE in different cell types. For example, whereas the Ras/MEK/ERK1/2/NF-*κ*B pathway plays a major role in (low) S100B/RAGE-induced neuronal survival (via upregulation of the antiapoptotic factor, Bcl-2) and (high) S100B/RAGE-induced neuronal death (via overproduction of ROS) [68], its activation is not critical for (high) S100B to upregulate the expression of the proinflammatory enzyme, COX-2, in microglia, S100B/RAGE activating a Cdc42/Rac1/JNK/AP-1 pathway and a Ras/Rac1/NF-*κ*B pathway in this latter case [64, 66]. Yet, the MEK/ERK1/2 pathway does mediate the S100B/RAGE-induced upregulation of IL-1*β* and TNF-*α* expression and secretion by microglia [84]. Incidentally, at low doses S100B does not activate microglia [60, 61], albeit synergizing with IL-1*β* and TNF-*α* to upregulate COX-2 expression [66]; instead, low S100B blocks trimethyltin-induced increase in TNF-*α* expression in microglia (via a mechanism that remains to be identified) [85]. Also, whereas high S100B has been shown to activate a RAGE/ROS/PI3K/Akt/NADPH oxidase/ROS pathway leading to lipid peroxidation and caspase-3 activation that cause dorsal root ganglia neuron apoptosis [86], the PI3K/Akt module does not appear to have any role in S100B/RAGE-induced upregulation of COX-2 expression in microglia [66]. Moreover, high levels of S100B cause GSK3*β*-dependent hyperphosphorylation of *τ* protein (a hallmark of Alzheimer disease) via RAGE-dependent activation of JNK and upregulation of Dickopff-1, a stimulator of GSK3*β* activity, in human neural stem cells [87]. So far, intermediates linking S100B/RAGE to signaling pathways include Src [88, 89] and diaphanous-1 [90]. It is tempting to speculate that the amount of RAGE expressed on the cell surface (which should condition the extent of RAGE oligomerization) and the relative fractions of octameric and multimeric S100B outside the cell might play a role in the choice between the intermediates recruited to RAGE, not to mention the potential recruitment to RAGE of other, cell-specific intermediates, possible crosstalks among intracellular signaling pathways, and autocrine/paracrine effects of factors released by the affected cells in consequence of the S100B/RAGE interaction.

Interestingly, levels of brain S100B in epileptic patients are increased compared with controls [91], and S100B release is increased in a mouse model of epilepsy [92]. In this latter experimental setting, the amplitude of hippocampal kainic acid-induced gamma oscillations is significantly reduced compared with WT mice, and released S100B enhances hippocampal kainic acid-induced gamma oscillations, an event that is abrogated by the local infusion of either an S100B neutralizing or a RAGE-neutralizing antibody [92]. Thus, S100B-activated RAGE signaling appears to make neurons more sensitive to the epileptogenic activity of kainic acid. Although no information is available about the cellular localization of RAGE in these studies, it is possible that S100B hyperpolarizes inhibitory interneurons in the hippocampus via RAGE engagement thereby causing dysinhibition of pyramidal neurons and enhancing their sensitivity to kainic acid. Indeed, some evidence suggests that S100B affects neuronal electrical discharge activity by modulation of potassium currents at low doses [93].

A role for S100B has been suggested in the pathogenesis and/or pathophysiology of schizophrenia based on the observation that serum levels of the protein are increased in this psychiatric disorder [94–96]. However, whereas in early studies the increased levels of S100B in patients with schizophrenia have been reported to occur without an indication for significant glial or neuronal damage, a finding that has been interpreted as an indirect evidence for increased active secretion of S100B by astrocytes during acute psychosis [97], other studies have shown that astrocyte and/or oligodendrocyte activation occurs in schizophrenic patients [98, 99]. Whereas alterations in glial and/or serum S100B levels may be indicative of participation of glial cells in the pathophysiology of schizophrenia, it is not known whether the increased serum S100B levels are indicative of the participation of the protein in the pathogenesis/pathophysiology of schizophrenia and what the role of (intracellular and/or extracellular) S100B in this psychiatric disorder might be. Recent work has shown that serum S100B levels normalize while levels of sRAGE (i.e., a product of digestion of RAGE acting as a scavenger of RAGE ligands) increase under antipsychotic treatment [100], suggesting that antipsychotic drugs might enhance the secretion/activity of matrix-metalloproteinases responsible for sRAGE production. Given the established role of RAGE in inflammation and of sRAGE as a protective factor against a number of inflammatory diseases [77, 78], and since a neuroinflammatory component characterizes psychotic states [101–103], one may hypothesize that during the course of acute schizophrenia activated astrocytes release more S100B either to aid in protecting neurons or to amplify neuroinflammation; the concomitant liberation of sRAGE from inflammatory cells (e.g., activated astrocytes and microglia) might then act to reduce the activity of RAGE ligands such as S100B, so as to extinguish/reduce the inflammatory response. However, this simplified model does not take into account the role of conventional cytokines and chemokines coming into play in the context of neuroinflammation and schizophrenia. In addition, enhancement of S100B release from astrocytes might not be causative of psychotic states per se because schizophrenia does not necessarily occur in Down syndrome (which is characterized by chronically elevated S100B levels (see [104, 105])), in aged people (who also show elevated S100B levels (see [106])), or in a variety of neuroinflammatory states characterized by elevated S100B levels [17]. Moreover, recent work raises the possibility that release of S100B from adipocytes contributes significantly to the elevated serum S100B levels found in schizophrenia [107, 108]. Although there is suggestion that variants within the S100B gene predispose to a psychotic subtype of bipolar affective disorder, possibly via alteration of gene expression [109–111], conclusions about the role of S100B in the pathogenesis/pathophysiology of schizophrenia should await more detailed analyses. The recent identification of *S100B* as a novel dyslexia candidate gene along with three other genes (i.e., *PCNT*, *DIP2A*, and *PRMT2*) mapping to chromosome region 21q22.3 suggests that decreases in S100B expression might contribute to certain dyslexia phenotypes [112]. This preliminary observation, however, lends support to the notion that alterations in S100B expression may have profound effects on brain functions and represents a further stimulus towards the elucidation of the functional role(s) of S100B at the cellular and molecular level.

Regulatory effects of extracellular S100B are not restricted to the brain. Aside from effects on monocytes/macrophages, neutrophils, myoblasts, and lens epithelial cells, S100B also exerts effects on vascular endothelial cells, vascular smooth muscle cells (VSMCs) (for review see [10]), and cardiomyocytes (see below). In fact, S100B engages RAGE in endothelial cells thereby activating NF-*κ*B transcriptional activity, increasing expression of vascular cell adhesion molecule-1, inducing monocyte chemoattractant protein-1 and RAGE transcripts and abrogating sodium nitroprusside-potentiated vasodilatation in response to ACh in endothelial dysfunction in type II diabetic (Lepr^db^) mice. Also, S100B enhances the interaction of RAGE with the leukocyte *β*2-integrin Mac-1, thus increasing leukocyte adhesion to endothelial cells. RAGE engagement by S100B causes VSMC proliferation (thus impacting on the pathogenesis of atherosclerosis) (Figure 1(c)) via stimulation of NADPH oxidase, increased ROS generation, and activation of phospholipase D2 and janus kinase (JAK) 2 tyrosine phosphorylation, these effects being enhanced in the presence of high glucose concentrations or angiotensin II. Moreover, S100B/RAGE interactions in VSMCs result in recruitment of the nonreceptor Src tyrosine kinase and PKC and phosphorylation of caveolin-1, a component of caveolae, which are stable membrane domains that are kept in place by the actin cytoskeleton and act as multifunctional organelles. Effects of S100B/RAGE on VSMCs and stimulation of VSMC migration and release of IL-6 (Figure 1(c)), require p38 MAPK, ERK1/2, NF-*κ*B and STAT3 activities, and ROS production. Recent evidence suggests that at concentrations >50 nM S100B induces cardiomyocyte apoptosis (Figure 1(d)) via RAGE-dependent phosphorylation of ERK1/2 and p53, increased expression and activity of proapoptotic caspase-3, and decreased expression of antiapoptotic Bcl-2 [113], another example of dissociation between intracellular and extracellular S100B effects.

### 3.2. Extracellular S100B: Just a DAMP Protein?

Most of the results commented on thus far point to S100B as to a damage-associated molecular pattern (DAMP) protein that is, a factor released from damaged/necrotic cells and endowed with the ability to activate cells of the innate immune response, alter the function of cell types (such as astrocytes, endothelial cells, and VSMCs) that participate in the inflammatory response, and/or cause cell death. In this regard, serum and cerebrospinal fluid levels of S100B are of diagnostic and/or prognostic value [17]. Compelling evidence suggests that extracellular S100B can be considered as a DAMP factor in the context of accumulation of the protein in the extracellular space: in this case, S100B might contribute twice to the inflammatory response, as a RAGE-activating ligand and as a factor capable of upregulating RAGE expression in reactive cells. Thus, S100B would contribute significantly to propagation of inflammation, and S100B-blocking agents might thus prove beneficial, attenuating inflammation and consequent cell damage.

However, differently from S100A8, S100A9, and S100A12, which are secreted by activated macrophages/neutrophils in response to inflammatory stimuli [114, 115], S100B is constitutively secreted by astrocytes in normal physiological conditions [49–53], and serum levels of S100B are relatively high at birth and in otherwise normal infants decreasing to picomolar levels around puberty [116]. Also, astrocytes might not be the sole source of serum S100B in normal and pathological conditions [49, 107, 108]. These observations suggest that the S100B's function outside the cell might go beyond its role as a DAMP protein and/or that DAMP proteins may also play a role in tissue development and/or regeneration. As mentioned earlier, at low doses, S100B protects neurons against apoptotic stimuli [68, 75, 117–119], enhances neurite outgrowth [120–125], and stimulates astrocyte proliferation [126], and the intraventricular infusion of low doses of S100B induces neurogenesis within the hippocampus, which is associated with an enhancement of cognitive functions following experimental traumatic brain injury [127, 128]. Also, the protein is released by in vitro trauma and reduces delayed neuronal injury [129–131]. The S100B protective effect towards neurons may also be indirect, the protein-stimulating uptake of the neurotoxic glutamate by astrocytes [132], reducing neurotoxin-dependent activation of microglia and astrocyte [85], protecting neurons against *β*-amyloid neurotoxicity [75] and reducing neuronal and glial cytotoxicity under hypothermic conditions [133]. Moreover, it has been suggested that the proliferation of neuronal precursors in the adult brain reported to occur following chronic treatment with antidepressants might be dependent on upregulation of S100B expression in astrocytes and RAGE expression in proliferating neuroblasts [134] although a causal relationship between upregulation of astrocytic S100B and neuronal RAGE in consequence of treatment with antidepressants and proliferation of neuronal precursors has not been established. Furthermore, extracellular regulatory effects of S100B might not be restricted to the brain, vasculature and heart, nor might they be dependent on RAGE exclusively. For example, nanomolar S100B stimulates myoblast proliferation, thus potentially contributing to the expansion of the myoblast population [71], a critical event during muscle development and regeneration [135]. It is intriguing, however, that RAGE and certain RAGE ligands including S100B might play important roles in such diverse contexts as the innate immune response on one side (see above) and tissue development and regeneration on the other side [8, 10, 68, 77, 78, 136–139]. Comparative analyses of wild-type, S100B KO, S100B TG, and RAGE KO mice might shed light in this regard.

Learning and memory processes long represent another field of action of extracellular S100B (for review see Refs. [1, 10, 140]). Indeed, S100B KO mice exhibit enhanced spatial and fear memories and enhanced long-term potentiation (LTP) in the hippocampal CA1 region, and perfusion of hippocampal slices with S100B reverses the levels of LTP to those of the wild-type slices [141]. This suggests that at physiological levels, extracellular S100B might play a role as a regulator of synaptic plasticity, although the molecular mechanism underlying this activity remains to be elucidated. Recently, hyperactivity and increased sensitivity to auditory stimuli have been reported in RAGE KO mice, with no significant differences between KO and wild types in behavioral tests for spatial memory and anxiety, though [142]. This preliminary evidence suggests that S100B/RAGE interactions might not be important for normal motor activity or spatial and fear memories, although definite conclusions should await analyses of double S100B-RAGE KO mice.

## 4. Conclusions

During the last two decades, the interest in S100B protein function in the brain and the cardiovascular apparatus has increased remarkably, mostly in view of its extracellular effects. By a combination of structural and functional studies, the emerging picture is one in which extracellular S100B exerts trophic and toxic effects depending on the concentration attained locally and the density of RAGE molecules expressed on the surface of responsive cells. Specifically, at low (i.e., nanomolar) S100B concentrations and in the presence of a relatively low RAGE cellular density, the protein might exert trophic effects, whereas at high (i.e., submicromolar-micromolar) and/or in the presence of a relatively high RAGE cellular density, the protein might be toxic, participating in the inflammatory response and causing cell death. In general, S100B behaves like a DAMP factor in a background of chronically elevated extracellular concentrations, like those occurring in Down syndrome, Alzheimer-like dementia, chronic neuroinflammation, atherosclerosis, and probably schizophrenia as well as whenever RAGE is found on the cell surface above a certain threshold of density. Thus, extracellular S100B effects appear to be context-dependent. However, S100B's trophic/toxic effects might not be necessarily transduced by RAGE [69–72, 119].

The great deal of information presented on regulatory effects of extracellular S100B has somewhat obscured the protein's intracellular function(s). The changes in the expression levels of S100B during certain phases of neural cell development in vivo and in vitro [22, 24], the enhanced S100B expression in reactive astrocytes (astrogliosis) [57] and gliomas (as well as in several nonnervous tumor cells) [4–6] and the involvement of intracellular S100B in cell proliferation and differentiation [12, 21–23] call for a detailed analysis of the regulation of S100B expression at the transcriptional and posttranscriptional level and of physiologically relevant interactions of S100B within cells. The importance of this issue is highlighted by the fact that: (1) the amount of released S100B generally is a small fraction of the protein's intracellular content (thus, the greater the content the larger the released fraction especially in the case of S100B leakage from damaged/necrotic cells); (2) within certain limits, intracellular regulatory effects are proportional to the S100B concentration within cells (see for example Refs. [18, 21, 22]); (3) the intracellular and extracellular effects of S100B are not univocal (as an example, at micromolar concentrations extracellular S100B cause astrocyte death via overproduction of NO [62] and neuronal and myoblast death via overproduction of ROS [68, 70]; yet, at the submicromolar-micromolar concentrations found within astrocytes and myoblasts S100B stimulates migration and, to a lesser extent, proliferation [22], and reduces differentiation via inhibition of MyoD expression [12], respectively); and (4) synthetic compounds with ability to block S100B activity such as arundic acid [38] and pentamidine [19] might not discriminate between intracellular and extracellular S100B. While further work is required for having a complete picture of intracellular and extracellular regulatory effects of S100B, current studies of the protein in physiological and pathological conditions are shedding light on the variety of cellular functions in which S100B is involved.

## References

[1] Donato R (2001). S100: a multigenic family of calcium-modulated proteins of the EF-hand type with intracellular and extracellular functional roles. *International Journal of Biochemistry and Cell Biology*.

[2] Marenholz I, Heizmann CW, Fritz G (2004). S100 proteins in mouse and man: from evolution to function and pathology (including an update of the nomenclature). *Biochemical and Biophysical Research Communications*.

[3] Heizmann CW, Fritz G, Bradshaw RA, Dennis EA (2009). The family of S100 cell signaling proteins. *Handbook of Cell Signaling*.

[4] Donato R (1991). Perspectives in S-100 protein biology. *Cell Calcium*.

[5] Donato R (1999). Functional roles of S100 proteins, calcium-binding proteins of the EF-hand type. *Biochimica et Biophysica Acta*.

[6] Heizmann CW, Fritz G, Schäfer BW (2002). S100 proteins: structure, functions and pathology. *Front Biosci*.

[7] Santamaria-Kisiel L, Rintala-Dempsey AC, Shaw GS (2006). Calcium-dependent and -independent interactions of the S100 protein family. *Biochemical Journal*.

[8] Donato R (2007). RAGE: a single receptor for several ligands and different cellular responses: the case of certain S100 proteins. *Current Molecular Medicine*.

[9] Leclerc E, Fritz G, Vetter SW, Heizmann CW (2009). Binding of S100 proteins to RAGE: an update. *Biochimica et Biophysica Acta*.

[10] Donato R, Sorci G, Riuzzi F (2009). S100B’s double life: intracellular regulator and extracellular signal. *Biochimica et Biophysica Acta*.

[11] Tsoporis JN, Overgaard CB, Izhar S, Parker TG (2009). S100B modulates the hemodynamic response to norepinephrine stimulation. *American Journal of Hypertension*.

[12] Tubaro C, Arcuri C, Giambanco I, Donato R (2010). S100B protein in myoblasts modulates myogenic differentiation via NF-*κ*B-dependent inhibition of MyoD expression. *Journal of Cellular Physiology*.

[13] Parker TG, Marks A, Tsoporis JN (1998). Induction of S100b in myocardium: an intrinsic inhibitor of cardiac hypertrophy. *Canadian Journal of Applied Physiology*.

[14] Tsoporis JN, Marks A, Van Eldik LJ, O’Hanlon D, Parker TG (2003). Regulation of the S100B gene by *α*1-adrenergic stimulation in cardiac myocytes. *American Journal of Physiology*.

[15] Tsoporis JN, Marks A, Haddad A, Dawood F, Liu PP, Parker TG (2005). S100B expression modulates left ventricular remodeling after myocardial infarction in mice. *Circulation*.

[16] Suzuki F, Kato K, Nakajima T (1984). Hormonal regulation of adipose S-100 protein release. *Journal of Neurochemistry*.

[17] Sen J, Belli A (2007). S100B in neuropathologic states: the CRP of the brain?. *Journal of Neuroscience Research*.

[18] Lin J, Blake M, Tang C (2001). Inhibition of p53 transcriptional activity by the S100B calcium-binding protein. *The Journal of Biological Chemistry*.

[19] Markowitz J, Chen I, Gitti R (2004). Identification and characterization of small molecule inhibitors of the calcium-dependent S100B-p53 tumor suppressor interaction. *Journal of Medicinal Chemistry*.

[20] Smith J, Stewart BJ, Glaysher S (2010). The effect of pentamidine on melanoma ex vivo. *Anti-Cancer Drugs*.

[21] Arcuri C, Bianchi R, Brozzi F, Donato R (2005). S100B increases proliferation in PC12 neuronal cells and reduces their responsiveness to nerve growth factor via Akt activation. *The Journal of Biological Chemistry*.

[22] Brozzi F, Arcuri C, Giambanco I, Donato R (2009). S100B protein regulates astrocyte shape and migration via interaction with Src Kinase: implications for astrocyte development, activation, and tumor growth. *The Journal of Biological Chemistry*.

[23] Saito T, Ikeda T, Nakamura K, Chung U-I, Kawaguchi H (2007). S100A1 and S100B, transcriptional targets of SOX trio, inhibit terminal differentiation of chondrocytes. *EMBO Reports*.

[24] Raponi E, Agenes F, Delphin C (2007). S100B expression defines a state in which GFAP-expressing cells lose their neural stem cell potential and acquire a more mature developmental stage. *Glia*.

[25] Rietze RL, Reynolds BA (2006). Neural stem cell isolation and characterization. *Methods in Enzymology*.

[26] Whitaker-Azmitia PM, Wingate M, Borella A, Gerlai R, Roder J, Azmitia EC (1997). Transgenic mice overexpressing the neurotrophic factor S-100*β* show neuronal cytoskeletal and behavioral signs of altered aging processes: implications for Alzheimer’s disease and Down’s syndrome. *Brain Research*.

[27] Esposito G, Imitola J, Lu J (2008). Genomic and functional profiling of human Down syndrome neural progenitors implicates S100B and aquaporin 4 in cell injury. *Human Molecular Genetics*.

[28] Sofroniew MV (2009). Molecular dissection of reactive astrogliosis and Glial scar formation. *Trends in Neurosciences*.

[29] Rommel C, Camps M, Ji H (2007). PI3K*δ* and PI3K*γ*: partners in crime in inflammation in rheumatoid arthritis and beyond?. *Nature Reviews Immunology*.

[30] Fruman DA, Bismuth G (2009). Fine tuning the immune response with PI3K. *Immunological Reviews*.

[31] Farina C, Aloisi F, Meinl E (2007). Astrocytes are active players in cerebral innate immunity. *Trends in Immunology*.

[32] Herrera F, Chen Q, Fischer WH, Maher P, Schubert DR (2009). Synaptojanin-1 plays a key role in astrogliogenesis: possible relevance for Down’s syndrome. *Cell Death and Differentiation*.

[33] Wu C-Y, Hsieh H-L, Sun C-C, Tseng C-P, Yang C-M (2008). IL-1*β* induces proMMP-9 expression via c-Src-dependent PDGFR/PI3K/Akt/p300 cascade in rat brain astrocytes. *Journal of Neurochemistry*.

[34] Ke Y, Jiang G, Sun D, Kaplan HJ, Shao H (2009). Retinal astrocytes respond to IL-17 differently than retinal pigment epithelial cells. *Journal of Leukocyte Biology*.

[35] Wang H-H, Hsieh H-L, Wu C-Y, Sun C-C, Yang C-M (2009). Oxidized low-density lipoprotein induces matrix metalloproteinase-9 expression via a p42/p44 and JNK-dependent AP-1 pathway in brain astrocytes. *Glia*.

[36] Wu J, Wrathall JR, Schachner M (2010). Phosphatidylinositol 3-Kinase/protein Kinase Cdelta activation induces close homolog of adhesion molecule L1 (CHL1) expression in cultured astrocytes. *Glia*.

[37] Kato H, Kurosaki R, Oki C, Araki T (2004). Arundic acid, an astrocyte-modulating agent, protects dopaminergic neurons against MPTP neurotoxicity in mice. *Brain Research*.

[38] Asano T, Mori T, Shimoda T (2005). Arundic acid (ONO-2506) ameliorates delayed ischemic brain damage by preventing astrocytic overproduction of S100B. *Current Drug Targets*.

[39] Mori T, Tan J, Arendash GW, Koyama N, Nojima Y, Town T (2008). Overexpression of human S100B exacerbates brain damage and periinfarct gliosis after permanent focal ischemia. *Stroke*.

[40] Wainwright MS, Craft JM, Griffin WST (2004). Increased susceptibility of S100B transgenic mice to peinatal hypoxia-ischemia. *Annals of Neurology*.

[41] Mori T, Koyama N, Arendash GW, Horikoshi-Sakuraba Y, Tan J, Town T (2010). Overexpression of human S100B exacerbates cerebral amyloidosis and gliosis in the Tg2576 mouse model of Alzheimer’s disease. *Glia*.

[42] Van Eldik LJ, Wainwright MS (2003). The Janus face of Glial-derived S100B: beneficial and detrimental functions in the brain. *Restorative Neurology and Neuroscience*.

[43] Xiong Z-G, O’Hanlon D, Becker LE, Roder J, MacDonald JF, Marks A (2000). Enhanced calcium transients in Glial cells in neonatal cerebellar cultures derived from S100B null mice. *Experimental Cell Research*.

[44] Haase H, Alvarez J, Petzhold D (1999). Ahnak is critical for cardiac Ca(V)1.2 calcium channel function and its beta-adrenergic regulation. *The FASEB Journal*.

[45] Lee IH, You JO, Ha KS (2004). AHNAK-mediated activation of phospholipase C-*γ*1 through protein Kinase C. *The Journal of Biological Chemistry*.

[46] Gentil BJ, Delphin C, Benaud C, Baudier J (2003). Expression of the giant protein AHNAK (desmoyokin) in muscle and lining epithelial cells. *Journal of Histochemistry and Cytochemistry*.

[47] Gentil BJ, Delphin C, Mbele GO (2001). The giant protein AHNAK is a specific target for the calcium-and zinc-binding S100B protein: potential implications for Ca^2+^ homeostasis regulation by S100B. *The Journal of Biological Chemistry*.

[48] Michetti F, Massaro A, Murazio M (1979). The nervous system-specific S-100 antigen in cerebrospinal fluid of multiple sclerosis patients. *Neuroscience Letters*.

[49] Shashoua VE, Hesse GW, Moore BW (1984). Proteins of the brain extracellular fluid: evidence for release of S-100 protein. *Journal of Neurochemistry*.

[50] Van Eldik LJ, Zimmer DB (1987). Secretion of S-100 from rat C6 glioma cells. *Brain Research*.

[51] Nardin P, Tortorelli L, Quincozes-Santos A (2009). S100B secretion in acute brain slices: modulation by extracellular levels of Ca^2+^ and K^+^. *Neurochemical Research*.

[52] de Souza DF, Leite MC, Quincozes-Santos A (2009). S100B secretion is stimulated by IL-1*β* in Glial cultures and hippocampal slices of rats: likely involvement of MAPK pathway. *Journal of Neuroimmunology*.

[53] Leite MC, Galland F, de Souza DF (2009). Gap junction inhibitors modulate S100B secretion in astrocyte cultures and acute hippocampal slices. *Journal of Neuroscience Research*.

[54] Ostendorp T, Leclerc E, Galichet A (2007). Structural and functional insights into RAGE activation by multimeric S100B. *The EMBO Journal*.

[55] Ma W, Lee SE, Guo J (2007). RAGE ligand upregulation of VEGF secretion in ARPE-19 cells. *Investigative Ophthalmology and Visual Science*.

[56] Kligman D, Marshak DR (1985). Purification and characterization of a neurite extension factor from bovine brain. *Proceedings of the National Academy of Sciences of the United States of America*.

[57] Marshak DR, Pesce SA, Stanley LC, Griffin WST (1992). Increased S100*β* neurotrophic activity in Alzheimer’s disease temporal lobe. *Neurobiology of Aging*.

[58] Fanò G, Mariggiò MA, Angelella P (1993). The S-100 protein causes an increase of intracellular calcium and death of PC12 cells. *Neuroscience*.

[59] Hu J, Ferreira A, Van Eldik LJ (1997). S100*β* induces neuronal cell death through nitric oxide release from astrocytes. *Journal of Neurochemistry*.

[60] Petrova TV, Hu J, Van Eldik LJ (2000). Modulation of Glial activation by astrocyte-derived protein S100B: differential responses of astrocyte and microGlial cultures. *Brain Research*.

[61] Adami C, Sorci G, Blasi E, Agneletti AL, Bistoni F, Donato R (2001). S100B expression in and effects on microGlia. *Glia*.

[62] Hu J, Castets F, Guevara JL, Van Eldiki LJ (1996). S100*β* stimulates inducible nitric oxide synthase activity and mRNA levels in rat cortical astrocytes. *The Journal of Biological Chemistry*.

[63] Lam AGM, Koppal T, Akama KT (2001). Mechanism of Glial activation by S100B: involvement of the transcription factor NF*κ*B. *Neurobiology of Aging*.

[64] Bianchi R, Adami C, Giambanco I, Donato R (2007). S100B binding to RAGE in microGlia stimulates COX-2 expression. *Journal of Leukocyte Biology*.

[65] Ponath G, Schettler C, Kaestner F (2007). Autocrine S100B effects on astrocytes are mediated via RAGE. *Journal of Neuroimmunology*.

[66] Bianchi R, Giambanco I, Donato R (2008). S100B/RAGE-dependent activation of microGlia via NF-*κ*B and AP-1. Co-regulation of COX-2 expression by S100B, IL-1*β* and TNF-*α*. *Neurobiology of Aging*.

[67] Hofmann MA, Drury S, Fu C (1999). RAGE mediates a novel proinflammatory axis: a central cell surface receptor for S100/calgranulin polypeptides. *Cell*.

[68] Huttunen HJ, Kuja-Panula J, Sorci G, Agneletti AL, Donato R, Rauvala H (2000). Coregulation of neurite outgrowth and cell survival by amphoterin and S100 proteins through receptor for advanced glycation end products (RAGE) activation. *The Journal of Biological Chemistry*.

[69] Sorci G, Riuzzi F, Agneletti AL, Marchetti C, Donato R (2003). S100B inhibits myogenic differentiation and myotube formation in a RAGE-independent manner. *Molecular and Cellular Biology*.

[70] Sorci G, Riuzzi F, Agneletti AL, Marchetti C, Donato R (2004). S100B causes apoptosis in a myoblast cell line in a RAGE-independent manner. *Journal of Cellular Physiology*.

[71] Riuzzi F, Sorci G, Donato R (2006). S100B stimulates myoblast proliferation and inhibits myoblast differentiation by independently stimulating ERK1/2 and inhibiting p38 MAPK. *Journal of Cellular Physiology*.

[72] Adami C, Bianchi R, Pula G, Donato R (2004). S100B-stimulated NO production by BV-2 microGlia is independent of RAGE transducing activity but dependent on RAGE extracellular domain. *Biochimica et Biophysica Acta*.

[73] Esposito G, De Filippis D, Cirillo C, Sarnelli G, Cuomo R, Iuvone T (2006). The astroGlial-derived S100*β* protein stimulates the expression of nitric oxide synthase in rodent macrophages through p38 MAP Kinase activation. *Life Sciences*.

[74] Omori K, Ohira T, Uchida Y (2008). Priming of neutrophil oxidative burst in diabetes requires preassembly of the NADPH oxidase. *Journal of Leukocyte Biology*.

[75] Businaro R, Leone S, Fabrizi C (2006). S100B protects LAN-5 neuroblastoma cells against A*β* amyloid-induced neurotoxicity via RAGE engagement at low doses but increases A*β* amyloid neurotoxicity at high doses. *Journal of Neuroscience Research*.

[76] Lue L-F, Walker DG, Brachova L (2001). Involvement of microGlial receptor for advanced glycation endproducts (RAGE)in Alzheimer’s disease: identification of a cellular activation mechanism. *Experimental Neurology*.

[77] Schmidt AM, Yan SD, Yan SF, Stern DM (2001). The multiligand receptor RAGE as a progression factor amplifying immune and inflammatory responses. *The Journal of Clinical Investigation*.

[78] Bierhaus A, Humpert PM, Morcos M (2005). Understanding RAGE, the receptor for advanced glycation end products. *Journal of Molecular Medicine*.

[79] Xie J, Reverdatto S, Frolov A, Hoffmann R, Burz DS, Shekhtman A (2008). Structural basis for pattern recognition by the receptor for advanced glycation end products (RAGE). *The Journal of Biological Chemistry*.

[80] Glezer I, Simard AR, Rivest S (2007). Neuroprotective role of the innate immune system by microGlia. *Neuroscience*.

[81] Hanisch U-K, Kettenmann H (2007). MicroGlia: active sensor and versatile effector cells in the normal and pathologic brain. *Nature Neuroscience*.

[82] Wainwright MS, Craft JM, Griffin WST (2004). Increased susceptibility of S100B transgenic mice to peinatal hypoxia-ischemia. *Annals of Neurology*.

[83] Mori T, Koyama N, Arendash GW, Horikoshi-Sakuraba Y, Tan J, Town T (2010). Overexpression of human S100B exacerbates cerebral amyloidosis and gliosis in the Tg2576 mouse model of Alzheimer’s disease. *Glia*.

[84] Kim SH, Smith CJ, Van Eldik LJ (2004). Importance of MAPK pathways for microGlial pro-inflammatory cytokine IL-1*β* production. *Neurobiology of Aging*.

[85] Reali C, Scintu F, Pillai R, Donato R, Michetti F, Sogos V (2005). S100B counteracts effects of the neurotoxicant trimethyltin on astrocytes and microGlia. *Journal of Neuroscience Research*.

[86] Vincent AM, Perrone L, Sullivan KA (2007). Receptor for advanced glycation end products activation injures primary sensory neurons via oxidative stress. *Endocrinology*.

[87] Esposito G, Scuderi C, Lu J (2008). S100B induces tau protein hyperphosphorylation via Dickopff-1 up-regulation and disrupts the Wnt pathway in human neural stem cells. *Journal of Cellular and Molecular Medicine*.

[88] Xu D, Kyriakis JM (2003). Phosphatidylinositol 3′-Kinase-dependent activation of renal mesangial cell Ki-Ras and ERK by advanced glycation end products. *The Journal of Biological Chemistry*.

[89] Reddy MA, Li S-L, Sahar S (2006). Key role of Src Kinase in S100B-induced activation of the receptor for advanced glycation end products in vascular smooth muscle cells. *The Journal of Biological Chemistry*.

[90] Hudson BI, Kalea AZ, Del Mar Arriero M (2008). Interaction of the RAGE cytoplasmic domain with diaphanous-1 is required for ligand-stimulated cellular migration through activation of Rac1 and Cdc42. *The Journal of Biological Chemistry*.

[91] Griffin WST, Yeralan O, Sheng JG (1995). Overexpression of the neurotrophic cytokine S100*β* in human temporal lobe epilepsy. *Journal of Neurochemistry*.

[92] Sakatani S, Seto-Ohshima A, Shinohara Y (2008). Neural-activity-dependent release of S100B from astrocytes enhances kainate-induced gmma oscillations in vivo. *Journal of Neuroscience*.

[93] Kubista H, Donato R, Hermann A (1999). S100 calcium binding protein affects neuronal electrical discharge activity by modulation of potassium currents. *Neuroscience*.

[94] Wiesmann M, Wandinger KP, Missler U (1999). Elevated plasma levels of S-100b protein in schizophrenic patients. *Biological Psychiatry*.

[95] Lara DR, Gama CS, Belmonte-de-Abreu P (2001). Increased serum S100B protein in schizophrenia: a study in medication-free patients. *Journal of Psychiatric Research*.

[96] Rothermundt M, Missler U, Arolt V (2001). Increased S100B blood levels in unmedicated and treated schizophrenic patients are correlated with negative symptomatology. *Molecular Psychiatry*.

[97] Steiner J, Walter M, Wunderlich MT (2009). A new pathophysiological aspect of S100B in schizophrenia: potential regulation of S100B by its scavenger soluble RAGE. *Biological Psychiatry*.

[98] Rothermundt M, Ohrmann P, Abel S (2007). Glial cell activation in a subgroup of patients with schizophrenia indicated by increased S100B serum concentrations and elevated myo-inositol. *Progress in Neuro-Psychopharmacology and Biological Psychiatry*.

[99] Steiner J, Bernstein H-G, Bielau H (2008). S100B-immunopositive Glia is elevated in paranoid as compared to residual schizophrenia: a morphometric study. *Journal of Psychiatric Research*.

[100] Steiner J, Walter M, Wunderlich MT (2009). A new pathophysiological aspect of S100B in schizophrenia: potential regulation of S100B by its scavenger soluble RAGE. *Biological Psychiatry*.

[101] Drzyzga Ł, Obuchowicz E, Marcinowska A, Herman ZS (2006). Cytokines in schizophrenia and the effects of antipsychotic drugs. *Brain, Behavior, and Immunity*.

[102] Potvin S, Stip E, Sepehry AA, Gendron A, Bah R, Kouassi E (2008). Inflammatory cytokine alterations in schizophrenia: a systematic quantitative review. *Biological Psychiatry*.

[103] Müller N (2010). COX-2 inhibitors as antidepressants and antipsychotics: clinical evidence. *Current Opinion in Investigational Drugs*.

[104] Allore R, O’Hanlon D, Price R (1988). Gene encoding the *β* subunit of S100 protein is on chromosome 21: implications for Down’s syndrome. *Science*.

[105] Mrak RE, Griffin WST (2004). Trisomy 21 and the brain. *Journal of Neuropathology and Experimental Neurology*.

[106] Sheng JG, Mrak RE, Rovnaghi CR, Kozlowska E, Van Eldik LJ, Griffin WST (1996). Human brain S100*β* and S100*β* mRNA expression increases with age: pathogenic implications for Alzheimer’s disease. *Neurobiology of Aging*.

[107] Steiner J, Walter M, Guest P (2010). Elevated S100B levels in schizophrenia are associated with insulin resistance. *Molecular Psychiatry*.

[108] Steiner J, Schiltz K, Walter M (2010). S100B serum levels are closely correlated with body mass index: an important caveat in neuropsychiatric research. *Psychoneuroendocrinology*.

[109] Liu J, Shi Y, Tang J (2005). SNPs and haplotypes in the S100B gene reveal association with schizophrenia. *Biochemical and Biophysical Research Communications*.

[110] Roche S, Cassidy F, Zhao C (2007). Candidate gene analysis of 21q22: support for S100B as a susceptibility gene for bipolar affective disorder with psychosis. *American Journal of Medical Genetics Part B*.

[111] Hohoff C, Ponath G, Freitag CM (2010). Risk variants in the S100B gene predict elevated S100B serum concentrations in healthy individuals. *American Journal of Medical Genetics Part B*.

[112] Poelmans G, Engelen JJM, Van Lent-Albrechts J (2009). Identification of novel dyslexia candidate genes through the analysis of a chromosomal deletion. *American Journal of Medical Genetics Part B*.

[113] Tsoporis JN, Izhar S, Leong-Poi H, Desjardins J-F, Huttunen HJ, Parker TG (2010). S100B interaction with the receptor for advanced glycation end products (RAGE): a novel receptor-mediated mechanism for myocyte apoptosis postinfarction. *Circulation Research*.

[114] Foell D, Wittkowski H, Roth J (2007). Mechanisms of disease: a “DAMP” view of inflammatory arthritis. *Nature Clinical Practice Rheumatology*.

[115] Su YL, Raftery MJ, Goyette J, Hsu K, Geczy CL (2009). Oxidative modifications of S100 proteins: functional regulation by redox. *Journal of Leukocyte Biology*.

[116] Portela LVC, Tort ABL, Schaf DV (2002). The serum S100B concentration is age dependent. *Clinical Chemistry*.

[117] Ahlemeyer B, Beier H, Semkova I, Schaper C, Krieglstein J (2000). S-100*β* protects cultured neurons against glutamate- and staurosporine-induced damage and is involved in the antiapoptotic action of the 5 HT1A-receptor agonist, Bay x 3702. *Brain Research*.

[118] Kögel D, Peters M, König H-G (2004). S100B potently activates p65/c-Rel transcriptional complexes in hippocampal neurons: clinical implications for the role of S100B in excitotoxic brain injury. *Neuroscience*.

[119] Pichiule P, Chavez JC, Schmidt AM, Vannucci SJ (2007). Hypoxia-inducible factor-1 mediates neuronal expression of the receptor for advanced glycation end products following hypoxia/ischemia. *The Journal of Biological Chemistry*.

[120] Winningham-Major F, Staecker JL, Barger SW, Coats S, Van Eldik LJ (1989). Neurite extension and neuronal survival activities of recombinant S100*β* proteins that differ in the content and position of cysteine residues. *Journal of Cell Biology*.

[121] Haglid KG, Yang Q, Hamberger A, Bergman S, Widerberg A, Danielsen N (1997). S-100*β* stimulates neurite outgrowth in the rat sciatic nerve grafted with acellular muscle transplants. *Brain Research*.

[122] Van Eldik LJ, Christie-Pope B, Bolin LM, Shooter EM, Whetsell WO (1991). Neurotrophic activity of S-100*β* in cultures of dorsal root ganGlia from embryonic chick and fetal rat. *Brain Research*.

[123] Bhattacharyya A, Oppenheim RW, Prevette D, Moore BW, Brackenbury R, Ratner N (1992). S100 is present in developing chicken neurons and Schwann cells and promotes motor neuron survival in vivo. *Journal of Neurobiology*.

[124] Nishi M, Whitaker-Azmitia PM, Azmitia EC (1996). Enhanced synaptophysin immunoreactivity in rat hippocampal culture by 5-HT1A agonist, S100b, and corticosteroid receptor agonists. *Synapse*.

[125] Ueda S, Leonardi ETK, Bell J, Azmitia EC (1995). Serotonergic sprouting into transplanted C-6 gliomas is blocked by S-100*β* antisense gene. *Molecular Brain Research*.

[126] Selinfreund RH, Barger SW, Pledger WJ, Van Eldik LJ (1991). Neurotrophic protein S100*β* stimulates Glial cell proliferation. *Proceedings of the National Academy of Sciences of the United States of America*.

[127] Kleindienst A, McGinn MJ, Harvey HB, Colello RJ, Hamm RJ, Bullock MR (2005). Enhanced hippocampal neurogenesis by intraventricular S100B infusion is associated with improved cognitive recovery after traumatic brain injury. *Journal of Neurotrauma*.

[128] Kleindienst A, Bullock MR (2006). A critical analysis of the role of the neurotrophic protein S100B in acute brain injury. *Journal of Neurotrauma*.

[129] Ellis EF, Willoughby KA, Sparks SA, Chen T (2007). S100B protein is released from rat neonatal neurons, astrocytes, and microGlia by in vitro trauma and anti-S100 increases trauma-induced delayed neuronal injury and negates the protective effect of exogenous S100B on neurons. *Journal of Neurochemistry*.

[130] Willoughby KA, Kleindienst A, Müller C, Chen T, Muir JK, Ellis EF (2004). S100B protein is released by in vitro trauma and reduces delayed neuronal injury. *Journal of Neurochemistry*.

[131] Ramos AJ, Rubio MD, Defagot C, Hischberg L, Villar MJ, Brusco A (2004). The 5HT1A receptor agonist, 8-OH-DPAT, protects neurons and reduces astroglial reaction after ischemic damage caused by cortical devascularization. *Brain Research*.

[132] Tramontina F, Tramontina AC, Souza DF (2006). Glutamate uptake is stimulated by extracellular S100B in hippocampal astrocytes. *Cellular and Molecular Neurobiology*.

[133] Schmitt KRL, Kern C, Lange PE, Berger F, Abdul-Khaliq H, Hendrix S (2007). S100B modulates IL-6 release and cytotoxicity from hypothermic brain cells and inhibits hypothermia-induced axonal outgrowth. *Neuroscience Research*.

[134] Manev H, Uz T, Manev R (2003). Glia as a putative target for antidepressant treatments. *Journal of Affective Disorders*.

[135] Chargé SBP, Rudnicki MA (2004). Cellular and molecular regulation of muscle regeneration. *Physiological Reviews*.

[136] Rauvala H, Rouhiainen A (2010). Physiological and pathophysiological outcomes of the interactions of HMGB1 with cell surface receptors. *Biochimica et Biophysica Acta*.

[137] Sorci G, Riuzzi F, Arcuri C, Giambanco I, Donato R (2004). Amphoterin stimulates myogenesis and counteracts the antimyogenic factors basic fibroblast growth factor and S100B via RAGE binding. *Molecular and Cellular Biology*.

[138] Riuzzi F, Sorci G, Donato R (2006). The amphoterin (HMGB1)/receptor for advanced glycation end products (RAGE) pair modulates myoblast proliferation, apoptosis, adhesiveness, migration, and invasiveness: functional inactivation of RAGE in l6 myoblasts results in tumor formation in vivo. *The Journal of Biological Chemistry*.

[139] Riuzzi F, Sorci G, Donato R (2007). RAGE expression in rhabdomyosarcoma cells results in myogenic differentiation and reduced proliferation, migration, invasiveness, and tumor growth. *American Journal of Pathology*.

[140] Gerlai R, Roder J (1996). Spatial and nonspatial learning in mice: effects of S100*β* overexpression and age. *Neurobiology of Learning and Memory*.

[141] Nishiyama H, Knöpfel T, Endo S, Itohara S (2002). Glial protein S100B modulates long-term neuronal synaptic plasticity. *Proceedings of the National Academy of Sciences of the United States of America*.

[142] Sakatani S, Yamada K, Homma C (2009). Deletion of RAGE causes hyperactivity and increased sensitivity to auditory stimuli in mice. *PloS One*.

